# Nontraumatic Myositis Ossificans Adjacent to the Femoral Nerve: A Ubiquitin-Specific Peptidase 6 (USP6)-Rearranged Lesion Mimicking Malignancy

**DOI:** 10.7759/cureus.116056

**Published:** 2026-09-10

**Authors:** Evan T Keister, Saeed Asiry, Griffin Thomas, Burke Gao, Armaan Shah, Pamela Unger, Sapir Lev, Puneet Pawha, John A Boockvar

**Affiliations:** 1 Neurosurgery, Northwell, New Hyde Park, USA; 2 Department of Neurosurgery, Lenox Hill Hospital, Donald and Barbara Zucker School of Medicine at Hofstra/Northwell, New York, USA; 3 Pathology, Northwell, New Hyde Park, USA; 4 Department of Pathology, Lenox Hill Hospital, Donald and Barbara Zucker School of Medicine at Hofstra/Northwell, New York, USA; 5 Orthopedic Surgery, Northwell, New Hyde Park, USA; 6 Department of Orthopedic Surgery, Lenox Hill Hospital, Donald and Barbara Zucker School of Medicine at Hofstra/Northwell, New York, USA; 7 Radiology, Northwell, New Hyde Park, USA; 8 Department of Radiology, Lenox Hill Hospital, Donald and Barbara Zucker School of Medicine at Hofstra/Northwell, New York, USA

**Keywords:** femoral nerve compression, heterotopic ossification, myositis ossificans, non-traumatic lesion, usp6 gene rearrangement

## Abstract

Myositis ossificans (MO) is a benign, self-limiting lesion characterized by heterotopic ossification that can mimic malignancy both radiographically and histologically. Non-traumatic MO is particularly challenging to diagnose, especially when arising adjacent to major neurovascular structures. Recent studies have identified ubiquitin-specific peptidase 6 (USP6) gene rearrangements in a subset of MO, linking it to a family of transient fibroblastic/myofibroblastic neoplasms. We present a 21-year-old female who presented with progressive right lower extremity pain and weakness without a history of trauma. Imaging revealed an intramuscular thigh mass adjacent to the femoral nerve, raising concern for a peripheral nerve sheath tumor or malignancy. Due to worsening symptoms and diagnostic uncertainty, the patient underwent surgical resection with femoral nerve neuroplasty. Histopathological analysis demonstrated a spindle cell lesion with zonal ossification, and fluorescence in situ hybridization confirmed a USP6 gene rearrangement, establishing the diagnosis of non-traumatic MO. Postoperatively, the patient experienced improvement in strength and partial sensory recovery. Non-traumatic MO can closely mimic peripheral nerve sheath tumors when arising adjacent to major nerves, creating significant diagnostic uncertainty. This case highlights the importance of integrating imaging features, histology, and molecular confirmation of USP6 rearrangement to accurately distinguish benign pseudosarcomatous lesions from malignancy and guide appropriate management.

## Introduction

Myositis ossificans (MO) is a benign, self-limiting lesion characterized by heterotopic ossification that typically presents as a solitary focus within soft tissue compartments, including tendons, nerves, and subcutaneous fat [1]. The pathophysiology of MO remains incompletely understood, but it is thought to involve inappropriate differentiation of fibroblasts into osteogenic cells, driven by dysregulated local stem cell activity following tissue injury, overuse, or genetic predisposition [1,2]. Historically, MO was classified into three subgroups defined by etiology: (1) MO progressiva (fibrodysplasia ossificans progressiva), resulting from a mutation of the activin A receptor type-1 (ACVR1) protein; (2) circumscribed MO, occurring with direct trauma; and (3) non-traumatic MO, occurring without a history of trauma [2,3]. Histologically, most MO lesions exhibit a distinct zonal architecture with a central immature osteoid region that transitions to outer mature spongy bone. This zonation helps distinguish MO from sarcomas, which often show a reverse or disorganized zonation pattern [4]. Notably, MO can histologically resemble malignancy, risking overdiagnosis and overtreatment [4,5].

Recent molecular studies have demonstrated that MO is part of a broader family of transient, self-limiting fibroblastic/myofibroblastic neoplasms, including aneurysmal bone cysts (ABCs), nodular fasciitis (NF), and fibro-osseous pseudotumor of the digits (FOPD), that frequently harbor rearrangements of the ubiquitin-specific peptidase 6 (USP6) gene [5,6]. Among these, MO and FOPD share collagen type I alpha 1 chain-ubiquitin specific peptidase 6 (COL1A1-USP6) as the most common fusion variant, whereas NF is dominated by myosin heavy chain 9-ubiquitin specific peptidase 6 (MYH9-USP6) fusion. This genetic overlap underscores a shared neoplastic mechanism among these benign pseudosarcomatous lesions [5,6]. Since MO progressiva (fibrodysplasia ossificans progressiva) from the historical classification shows an ACVR1 mutation and not a USP6 gene rearrangement, it is considered a separate entity with systemic manifestations.

Peripheral nerve involvement is a recognized manifestation of MO, with reported cases involving major nerves including the sciatic, ulnar, median, brachial plexus, and femoral nerves [7-11]. Regarding femoral nerve involvement, Druce et al. (2002) [10] described a case of MO progressiva and Lee et al. (2013) [11] reported MO circumscripta, although both without molecular characterization confirming USP6 rearrangement.

Here, we present the case of a previously healthy 21-year-old female with non-traumatic myositis ossificans, harboring a confirmed USP6 gene rearrangement, arising adjacent to the femoral nerve and radiographically mimicking a peripheral nerve sheath tumor. This case represents, to our knowledge, the first molecularly confirmed example of USP6-rearranged non-traumatic MO presenting with femoral nerve compression, expanding the clinical spectrum of USP6-associated myofibroblastic lesions.

## Case presentation

A 21-year-old female with no significant past medical history presented with a two-week history of worsening right lower extremity pain radiating from the hip to the shin, without antecedent trauma or another identifiable precipitating event, including recent surgery, intramuscular injection, or strenuous exercise. She reported associated numbness and tingling, describing the initial discomfort as a sensation of stiffness that limited her ability to exercise. The pain progressed and rendered the patient unable to walk, pursue school, hobbies, and activities of daily living. Neurological examination revealed mild right lower extremity weakness. Medical Research Council (MRC) scale was M3 in the iliopsoas and M4 in hip flexion, hip extension, knee extension, knee flexion, tibialis anterior, extensor hallucis longus, and gastrocnemius. Strength in the remaining muscle groups was M5. Sensory examination demonstrated preserved light touch corresponding to S4 on the ASIA sensory scale throughout the right lower extremity, and deep tendon reflexes were symmetric.

MRI revealed a right proximal thigh lesion adjacent to the femoral nerve measuring 2.3 x 2.5 x 4.1 cm (Figure 1). The initial differential diagnosis included a spontaneous hematoma versus a peripheral nerve sheath tumor versus myositis ossificans. Meloxicam (7.5 mg daily), acetaminophen (1,000 mg three times daily), and methylprednisolone (Medrol®) 4 mg taper pack were administered for pain management but provided minimal relief.

**Figure 1:**
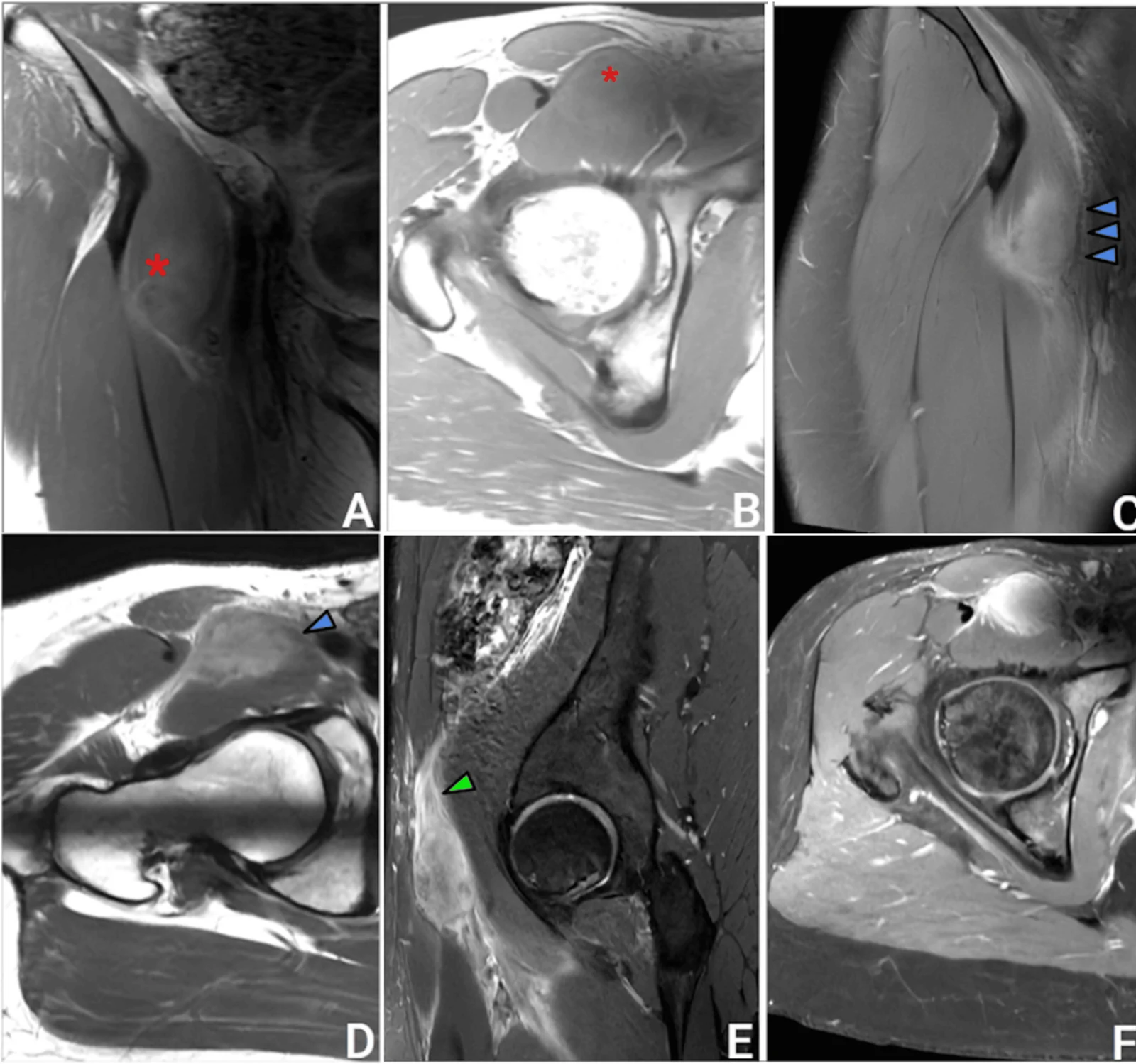
Preoperative MRI of nontraumatic myositis ossificans adjacent to the femoral nerve Coronal (A) and axial (B) T1-weighted images demonstrate a large intramuscular mass (red asterisk) in the right proximal thigh adjacent to the iliacus muscle. Coronal (C) and axial proton density (D) images show diffuse hyperintensity with surrounding soft-tissue edema and mild mass effect on the femoral neurovascular bundle; the femoral nerve (blue arrowhead) is displaced medially. Sagittal T2-weighted image (E) demonstrates a striated pattern of hyperintensity aligned with muscle fibers (green arrowhead). Axial contrast-enhanced T1-weighted image (F) shows diffuse enhancement without a discrete capsule.

Given the patient’s persistent and severe pain and to rule out malignancy in the differential diagnosis, a resection of the right thigh mass with femoral nerve neuroplasty was pursued. She was positioned supine, neuromonitoring was established, and a longitudinal incision was made over the palpable mass. The sartorius muscle was identified, its fascia and sheath were opened, and the muscle was mobilized medially to expose the rectus femoris. A firm, calcified mass measuring approximately 4 x 3 cm was found compressing branches of the femoral nerve and was excised in marginal fashion with preservation of the femoral neurovascular bundle. Gross total resection of the lesion was achieved.

Frozen section analysis revealed an intramuscular spindle cell lesion with bone formation. Permanent sections confirmed these findings, demonstrating a well-circumscribed proliferation of myofibroblastic spindle cells embedded within a matrix of immature bone and osteoid. Immunohistochemical staining for smooth muscle actin (SMA) was weakly positive in the spindle component, supporting myofibroblastic differentiation. An increased number of mitotic figures was noted, which is an expected finding in this entity (Figure 2). Given the spindle cell proliferation with associated bone and osteoid formation, molecular testing for USP6 rearrangement was performed because this alteration has been identified in MO and related benign fibroblastic/myofibroblastic lesions and can provide ancillary diagnostic support [5,6]. Fluorescence in situ hybridization (FISH) demonstrated a USP6 (17p13) gene rearrangement, supporting the diagnosis of MO.

**Figure 2:**
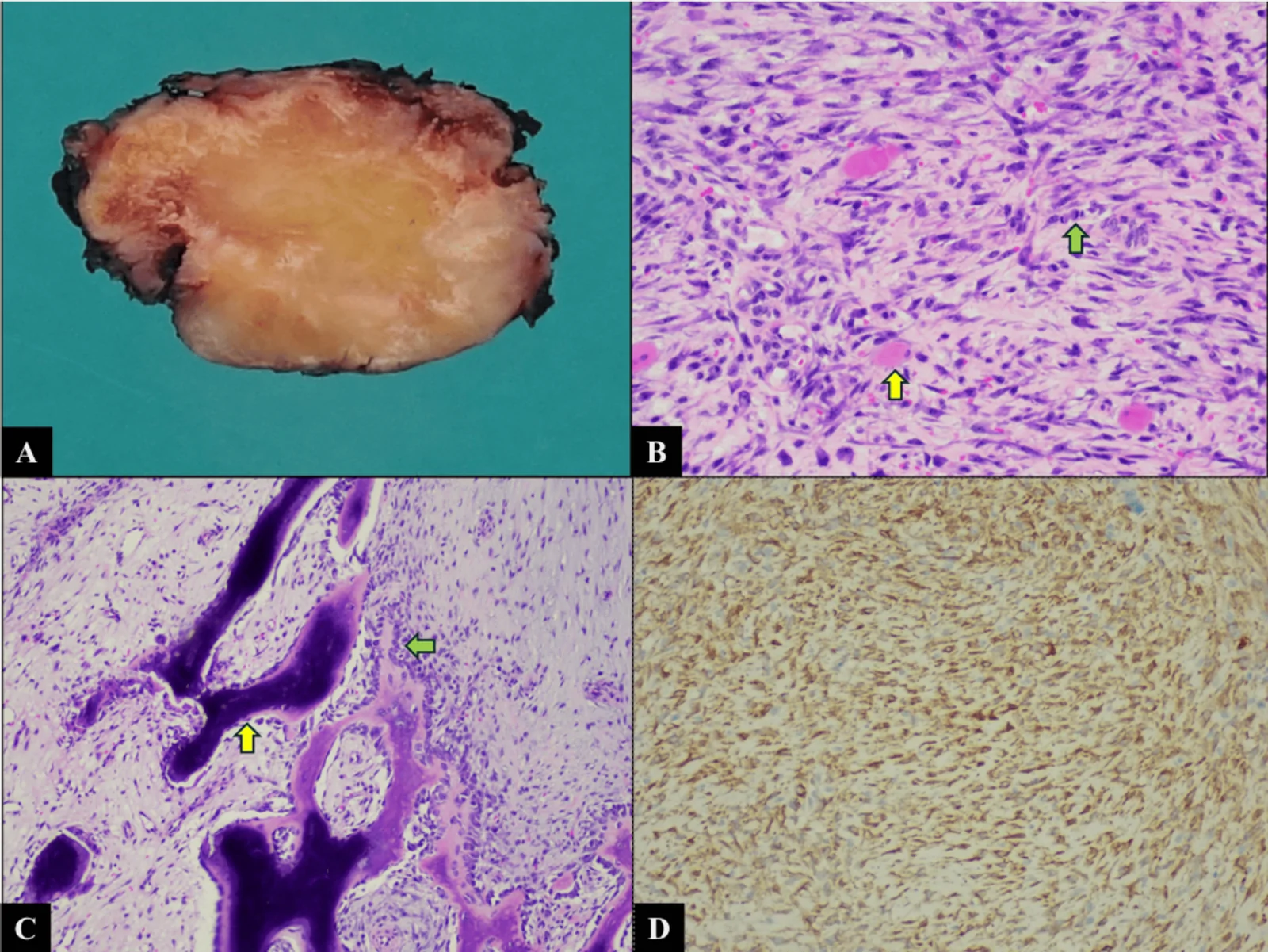
Gross and histopathologic features of USP6-rearranged myositis ossificans Gross photo of the cut section of the mass with tan-white to tan-pink smooth to faintly whorled, and glistening appearance with some areas of red-brown granularity (A). Hematoxylin and eosin-stained sections of the lesion with spindle cell proliferation seen between skeletal muscle fibers (yellow arrow) and easily identifiable mitotic figures (green arrow) (B, 200X). The periphery of the lesion showed woven bone formation (yellow arrow) with some reactive-appearing osteoblasts (green arrow) (C, 100X). By immunohistochemistry, the spindle cells showed weak positive labeling with SMA immune stain supporting their myofibroblastic differentiation (D, 200X).

Postoperatively, the patient reported intermittent “zapping” sensations in the right heel, which subsequently resolved. At the three-week follow-up visit, motor examination demonstrated improvement in strength, with M4- in hip flexion, M4 in knee extension, M4+ in extensor hallucis longus, and M5 in hip extension, knee flexion, tibialis anterior, and gastrocnemius. Sensory examination using the ASIA sensory scale revealed S3+ sensation in the L2-L3 dermatomes, with partial sensory recovery in peripheral nerve distributions: approximately 7/10 in the lateral femoral cutaneous nerve distribution, 3/10 in the medial femoral cutaneous distribution, and 8/10 along the lateral leg to the distal knee. Light touch remained preserved throughout the remainder of the extremity. The patient was ambulating independently with a mild antalgic gait, with subjective weakness.

## Discussion

This case highlights the diagnostic complexity of non-traumatic myositis ossificans (MO) presenting as a nerve-adjacent mass in a young adult with progressive neuropathic symptoms and functional impairment. To our knowledge, we identified no previously reported case of non-traumatic myositis ossificans with a demonstrated USP6 rearrangement presenting with femoral nerve compression. Although MO most commonly occurs within the thigh musculature, prior reports of femoral nerve involvement have been limited to MO progressiva and circumscripta without molecular confirmation of USP6 rearrangement [6,10-12]. This case therefore expands the anatomic and clinical spectrum of USP6-associated myofibroblastic lesions and demonstrates that non-traumatic MO can arise near the femoral nerve and present with compressive neuropathic symptoms that may clinically and radiographically mimic a peripheral nerve sheath tumor.

MO typically progresses through overlapping early, intermediate, and mature stages, beginning with inflammation and pain and evolving toward progressive ossification over several months [1]. Although frequently associated with antecedent injury, MO can also arise spontaneously in the absence of identifiable trauma [2,3]. This presentation can create substantial diagnostic uncertainty, particularly during the early stages of lesion development when characteristic mineralization may not yet be apparent. Non-traumatic MO therefore remains an important diagnostic consideration when evaluating a rapidly developing soft-tissue mass, even when there is no history of trauma.

The histopathologic appearance of MO can further complicate diagnosis. Early lesions may demonstrate hypercellular spindle cell proliferation and increased mitotic activity that can be mistaken for a malignant mesenchymal neoplasm, particularly in limited or early biopsy specimens [4,13]. The characteristic zonal maturation pattern, consisting of more mature peripheral bone surrounding a less organized central region, remains an important feature distinguishing MO from malignancy [4,13]. In the present case, histopathologic examination demonstrated a well-circumscribed proliferation of myofibroblastic spindle cells associated with immature bone and osteoid formation. Molecular testing by fluorescence in situ hybridization subsequently demonstrated a USP6 rearrangement, which, in conjunction with the morphologic and radiographic findings, supported the diagnosis of MO.

Contemporary molecular studies have further refined the understanding of MO by demonstrating its relationship to a broader family of USP6-rearranged fibroblastic and myofibroblastic neoplasms. These include nodular fasciitis, fibro-osseous pseudotumor of the digits, and aneurysmal bone cyst, among other lesions [4-6]. The identification of recurrent USP6 rearrangements in MO supports its relationship to these transient neoplastic processes and provides an additional molecular tool for distinguishing these lesions from malignant mimics [4-6]. In diagnostically challenging cases, integration of molecular findings with the characteristic histopathologic architecture and radiographic evolution may therefore improve diagnostic confidence.

From a clinical standpoint, this case also emphasizes the importance of maintaining a broad differential diagnosis for masses arising adjacent to major peripheral nerves. Although MO is benign and frequently self-limiting, surgical intervention may be warranted in patients with persistent or refractory pain, progressive neurologic compression, or substantial functional impairment [1,12]. In this patient, progressive pain, weakness, inability to ambulate normally, and continued diagnostic uncertainty regarding malignancy prompted surgical resection with femoral nerve neuroplasty. The lesion was excised while preserving the femoral neurovascular bundle, followed by improvement in motor function and partial sensory recovery.

The timing of surgical intervention in MO should be considered in the context of the lesion's etiology and clinical presentation. Historically, excision of traumatic MO has often been delayed until lesion maturation because of concern for recurrence. However, early excision of non-traumatic MO does not appear to carry the same degree of recurrence risk [13]. When a lesion causes significant neurologic compromise or persistent functional limitation, delaying intervention solely to permit radiographic maturation may therefore not always be appropriate [13]. Surgical management should instead be individualized according to symptom severity, anatomic relationships, diagnostic uncertainty, and the potential morbidity of continued compression.

This report is limited by its single-case design and relatively short postoperative follow-up. Nevertheless, the combination of an unusual anatomic presentation, femoral nerve compression, and molecular confirmation of USP6 rearrangement adds to the existing literature regarding the clinical spectrum of non-traumatic MO. Recognition of this entity is particularly relevant when evaluating nerve-adjacent soft-tissue masses in young patients, in whom an apparently aggressive clinical or radiographic presentation may otherwise raise concern for peripheral nerve sheath tumor or soft-tissue malignancy.

## Conclusions

Non-traumatic myositis ossificans can present as a symptomatic nerve-adjacent mass and closely mimic peripheral nerve sheath tumors or other soft-tissue neoplasms. This case adds to the reported spectrum of the recognized clinical and anatomic spectrum of USP6-rearranged myositis ossificans by demonstrating femoral nerve compression in the absence of antecedent trauma. Accurate diagnosis requires integration of the clinical presentation, radiographic features, histopathologic architecture, and, when appropriate, molecular findings. Recognition of this benign entity may help prevent misdiagnosis and unnecessary aggressive treatment while allowing timely surgical intervention when progressive neurologic compression, refractory pain, or functional impairment is present.
